# Ferroptosis Related Genes in Ischemic and Idiopathic Cardiomyopathy: Screening for Potential Pharmacological Targets

**DOI:** 10.3389/fcell.2022.817819

**Published:** 2022-03-03

**Authors:** Yufeng Jiang, Ling Chen, Zhujun Chao, Tan Chen, Yafeng Zhou

**Affiliations:** ^1^ Department of Cardiology, Suzhou Dushu Lake Hospital, Dushu Lake Hospital Affiliated to Soochow University, Medical Center of Soochow University, Suzhou, China; ^2^ Department of Endocrinology, The First Affiliated Hospital of Soochow University, Suzhou, China; ^3^ Suzhou Medical College of Soochow University, Suzhou, China

**Keywords:** ferroptosis, cardiomyopathy, precision medicine, ischemic, idiopathic

## Abstract

**Background:** Ferroptosis is a new form of cell death recently discovered that is distinct from apoptosis, necrosis and autophagy. This article is expected to provide a new direction for the treatment of cardiomyopathy in the future by screening potential drug targets associated with ferroptosis.

**Methods:** Differential expression analysis of GSE5406 from the Gene Expression Omnibus (GEO) database was performed using the GEO2R tool. Functional annotation of ferroptosis related genes was also performed. Then we constructed protein-protein interaction networks and identified hub genes using Cytoscape. The candidates for pharmacological compounds targeting the hub genes were screened by cMap.

**Results:** Totally 15 ferroptosis related genes (4 upregulated and 11 downregulated) for ischemic cardiomyopathy and 17 ferroptosis related genes (13 upregulated and 4 downregulated) for idiopathic cardiomyopathy were found. The biological processes involved in these genes mainly include negative regulation of apoptotic process, flavonoid metabolic process, response to drug for ischemic cardiomyopathy and cellular response to fibroblast growth factor stimulus, negative regulation of apoptotic process, and response to drug for idiopathic cardiomyopathy. KEGG results showed that these genes were mainly involved in MAPK signaling pathway for ischemic cardiomyopathy and PI3K-Akt signaling pathway for idiopathic cardiomyopathy. We generated a co-expression network for hub genes and obtained top 10 medications suggested respectively for ischemic/idiopathic cardiomyopathy.

**Conclusion:** Our study reveals the potential role of ferroptosis related genes in ischemic and idiopathic cardiomyopathy through bioinformatics analysis. The hub genes and potential drugs may become novel biomarkers for prognosis and precision treatment in the future.

## Introduction

Ischemic heart disease is defined as the presence of diffuse left ventricular systolic dysfunction resembling dilated cardiomyopathy due to coronary artery disease, including microcirculatory disturbance, resulting myocardial dystrophy with ischemia, degeneration, necrosis, fibrosis, and scarring of the myocardium (Sekulic et al., 2019; Halasz and Piepoli, 2020; Moroni et al., 2021). Idiopathic cardiomyopathy refers to a class of cardiomyopathies without an obvious pathogenic cause. It is characterized by marked enlargement of the left ventricle (vast majority) and/or right ventricle, thinning of the ventricular wall, and reduced ventricular systolic function, and it is characterized by heart enlargement, heart failure, severe arrhythmias and embolism, progressive disease progression, poor prognosis, and extremely high mortality (Ip and Lerman, 2018; Chen H. F., 2020). Cardiomyocyte is a terminally differentiated cell that is not renewable and can only be replaced by scar tissue once death occurs, thus triggering cardiac structure and dysfunction and eventually developing heart failure (Chen X. L et al., 2020).

Ferroptosis is a cell death modality characterized by iron dependence, excessive accumulation of lipid peroxides, regulated by several metabolic pathways and is morphologically, genetically and biochemically distinct from previous cell death modalities such as apoptosis, and pyroptosis or necrosis (Conrad and Proneth, 2019; Fang et al., 2019; Sumneang et al., 2020). Cardiomyocytes from patients with cardiomyopathy can undergo ferroptosis through both the canonical and noncanonical pathways (Chen et al., 2019; Park et al., 2019; Yin et al., 2020). Its pathophysiological mechanism is complex, involving oxidative stress, calcium overload, inflammation hyperactivation of inflammatory responses, increased release of excitatory amino acids, and apoptosis or necrosis, etc (Wu et al., 2021; Zhai et al., 2021). The discovery of cardiomyocyte ferroptosis in cardiomyopathy suggests that ferroptosis is a novel mechanism of cardiomyocyte injury, and targeted intervention of ferroptosis is expected to be an effective treatment for cardiomyopathy.

In this study, with the aid of bioinformatics, we searched for iron-related genes and pathways that are important for ischemic cardiomyopathy and idiopathic cardiomyopathy through the analysis of public databases, with the hope of finding potential therapeutic agents that could provide clues for further research and subsequent treatment of ischemic cardiomyopathy and idiopathic cardiomyopathy.

## Materials and Methods

### Source of Microarray Data

In this study, we downloaded a microarray dataset (GSE5406) from the Gene Expression Omnibus (GEO) database (https://www.ncbi.nlm.nih.gov/gds/) investigating gene expression profiles changes in patients with ischemic cardiomyopathy and idiopathic cardiomyopathy (Hannenhalli et al., 2006). The gene expression profile GSE5406 was generated based on the platform of GPL96 [HG-U133A] Affymetrix Human Genome U133A Array. This dataset contains myocardial biopsy findings from ischemic cardiomyopathy (*n* = 108), idiopathic cardiomyopathy (*n* = 86), and normal heart from organ donors (*n* = 16). Myocardial tissues were generally obtained from patients with advanced ischemic or idiopathic cardiomyopathy at the time of heart transplantation, or at the time of healthy heart donation. Left ventricular myocardium was snap-frozen for subsequent analyses.

### Sample Detection and Differential Expression genes Analysis

Volcano plots were drawn using fold change and corrected *p*-values. Boxplots were drawn by the R package ggplot2; Expression heatmaps were exhibited by the R package pheatmap. DEGs were analyzed using GEO2R (www.ncbi.nlm.nih.gov/geo/geo2r). The criteria for significance were adjusted *p* < 0.05aand |log2 fold change (FC)| >0.5.

### Ferroptosis Related Genes and Venn Analysis

GeneCards (https://www.Genecards.org/), BioGPS (http://biogps.org/), and Genehopper (http://genehopper.ifis.cs.tu-bs.de/) are databases integrating gene related functions and their experimental basis. We searched GeneCards, BioGPS, and Genehopper with “ferroptosis” as the key word for ferroptosis related genes. We used the Draw Venn Diagram (http://bioinformatics.psb.ugent.be/webtools/Venn/) tool to generate Venn diagrams between DEGs and ferroptosis related genes. DEGs that overlapped with ferroptosis related genes were included in subsequent analyses.

### Gene Ontology Enrichment Analysis and Kyoto Encyclopedia of Genes and Genomes Pathway Analysis

Gene ontology (GO) functional enrichment analysis (including analysis of molecular function [MF], biological process [BP], and cellular component [CC] terms) and Kyoto Encyclopedia of Genes and Genome (KEGG) pathway analysis of the DEGs were performed in the Database for Annotation, Visualization and Integrated Discovery (DAVID) v6.8 (http://david.ncifcrf.gov). *p* < 0.05 was the inclusion criterion.

### Protein-Protein Interaction Network Construction

The online database of STRING (https://www.string-db.org/) was used to analyze the DEGs to predict the interaction relationship between proteins encoded by genes that may play an important role in the pathogenesis of ischemic cardiomyopathy and idiopathic cardiomyopathy. Confidence interaction score was set at 0.15 for the significant criterion.

### Screening for Potential Pharmacological Targets

The cMAP (ConnectivityMap) database (https://clue.io/query) contains data on gene expression profile changes caused by 33,609 small molecule compounds acting on multiple cell lines, which can be used to compare similarities between drug-induced gene profiles and gene expression, and receives a connectivity score from −100 to 100: this score is greater than 0, which indicates similar changes caused and uploaded genes by compounds; A score less than 0 indicates that the compound causes and upload gene opposite changes, i.e., the compound may have a therapeutic effect on the disease. Small molecule compounds with connectivity scores <−80 as a result of promising prediction.

## Result

### Identification of Differentially Expressed Genes

**FIGURE 1:**
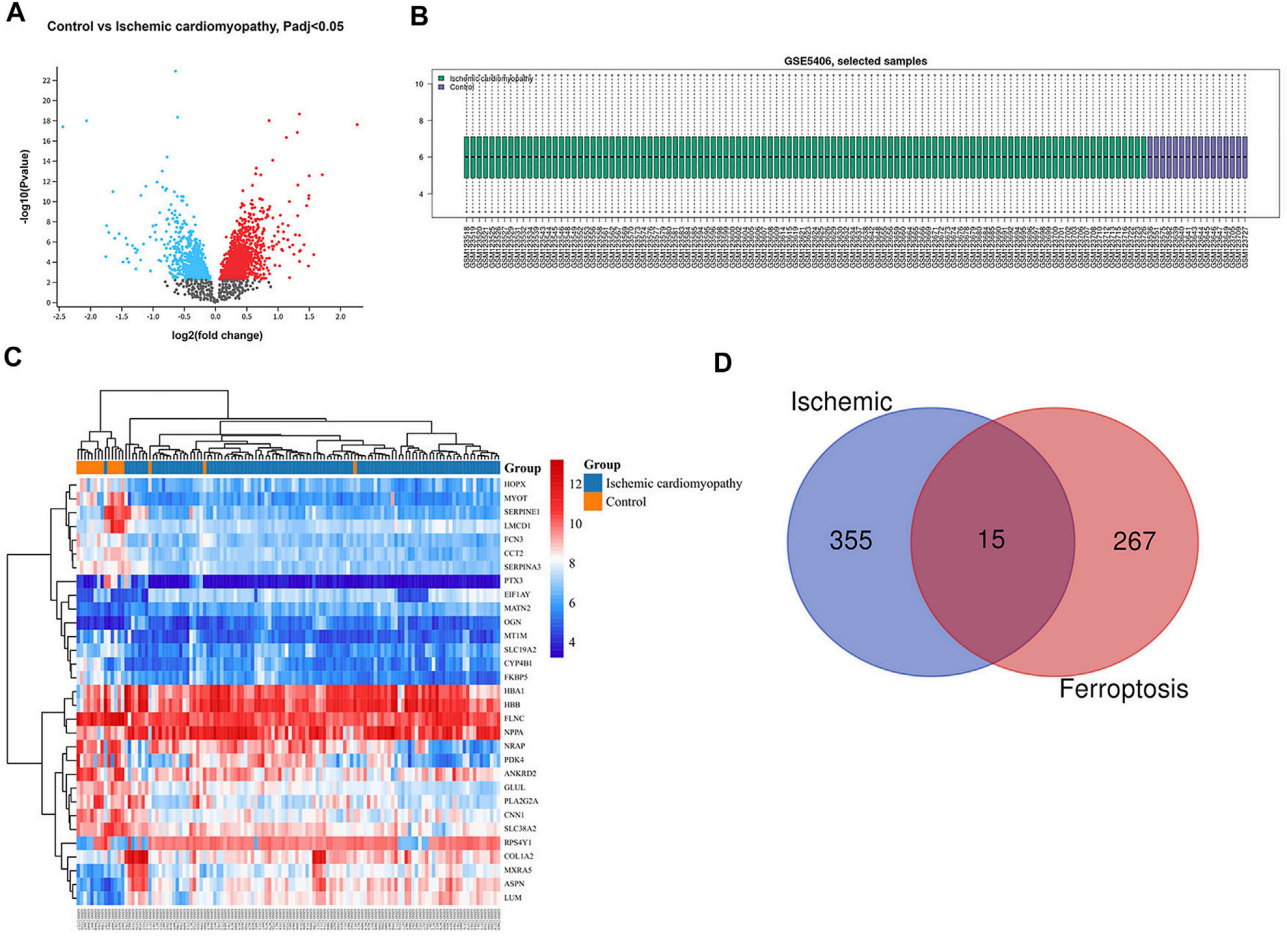
Differential expressed gene (DEG) analysis on ischemic cardiomyopathy. **(A)** Volcanic plots of gene expression of ischemic cardiomyopathy in GSE5406. Red represents upregulated DEGs, blue represents downregulated DEGs, grey represents genes which are not differentially expressed. **(B)** Cross comparability evaluation of microarray data. **(C)** Heat map of the DEGs. **(D)** Venn diagram of ferroptosis related DEGs.

**FIGURE 2:**
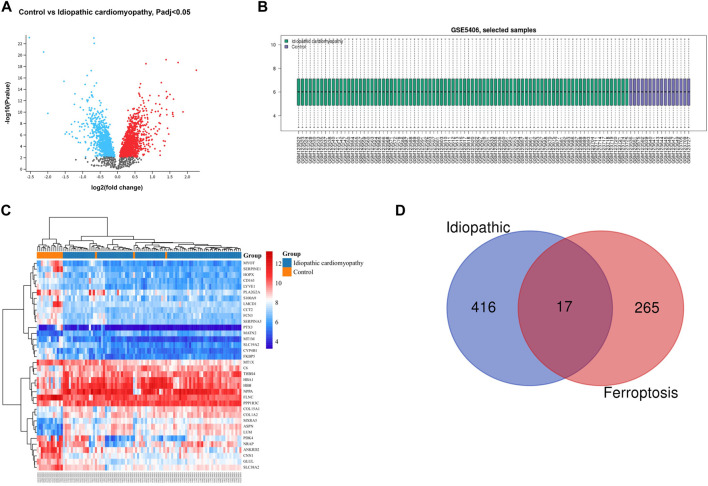
Differential expressed gene (DEG) analysis on idiopathic cardiomyopathy. **(A)** Volcanic plots of gene expression of idiopathic cardiomyopathy in GSE5406. **(B)** Cross comparability evaluation of microarray data. **(C)** Heat map of the DEGs. **(D)** Venn diagram of ferroptosis related DEGs.

The gene dataset GSE5406 contained a total of 108 ischemic cardiomyopathy samples, 86 idiopathic cardiomyopathy samples, and 16 normal myocardial tissue samples. Using the GEO2R online analysis tool for differential expression analysis, and using *p* < 0.05 and |log2 FC| > 0.5 as filtering conditions, we found that 370 genes were differentially expressed in the myocardial tissue of patients with ischemic cardiomyopathy compared with the normal myocardial tissue, of which 139 were up-regulated genes and 231 were down regulated genes, and clustering analysis of these differential genes was performed, as shown in the volcano plot (Figure 1A). A total of 433 genes were differentially expressed in the myocardium of patients with idiopathic cardiomyopathy, 215 of which were upregulated and 218 downregulated genes, and clustering analysis of these differential genes was performed, as shown in a volcano plot (Figure 2A). Data normalization and cross comparability were also assessed. As shown in Figures 1B, 2B, the selected samples were centered and numerically distributed up to standard, indicating the high quality and cross comparability of the microarray data. Figure 1C, 2C are heatmaps for the dataset, which indicates better clustering of samples and higher confidence.

### Ferroptosis Related Genes and Venn Analysis

Our search of GeneCards, BioGPS and Genehopper found 282 ferroptosis related target genes (Supplementary Table S1). By taking the intersection of the DEGs and ferroptosis related genes, 15 overlapped genes (MAP4, FZD7, MYH10, POR upregulated and HSPB1, FASN, HSPA9, EIF4A1, PLIN2, HSP90B1, AKR1C2, WTAP, MAP1LC3B, MYC, ATF4 downregulated) for ischemic cardiomyopathy (Figure 1D) and 17 overlapped genes (HSPB1, FASN, EIF4A1, PLIN2, HSP90B1, WTAP, YWHAZ, ZFP36, MAP1LC3B, POR, MYC, ATF4, SAT1 upregulated and MAP4, FZD7, MYH10, SNCA downregulated) for idiopathic cardiomyopathy (Figure 2D) were finally found to be included in the subsequent analyses.

### Gene Ontology (GO) Enrichment and Kyoto Encyclopedia of Genes and Genome (KEGG) Pathway Analysis

The gene ontology enrichment analysis of ferroptosis related differential genes contained three parts: biological process, cellular localization, and molecular function (Tables 1, 2). For ischemic cardiomyopathy (Table 1), the biological processes involved in these genes mainly include negative regulation of apoptotic process, flavonoid metabolic process, response to drug, regulation of translational initiation, and cellular response to UV; Genes are mainly located in extracellular matrix, mitochondrion, cytosol, extracellular exosome and focal adhesion; The main molecular functions are protein binding, poly(A) RNA binding and oxidoreductase activity. KEGG enrichment results showed that these genes were mainly involved in MAPK signaling pathway and HTLV-I infection (Table 3). For idiopathic cardiomyopathy (Table 2), the biological processes involved in these genes mainly include cellular response to fibroblast growth factor stimulus, response to drug, negative regulation of apoptotic process and so on; Genes are mainly located in cytosol, mitochondrion, melanosome etc.; The main molecular functions are protein binding, poly(A) RNA binding, oxidoreductase activity, microtubule binding and protein kinase binding. KEGG analysis indicated that these genes were mainly involved in PI3K-Akt signaling pathway, Hepatitis B, and Hippo signaling pathway (Table 3).

**TABLE 1:** Gene ontology enrichment analysis for ischemic cardiomyopathy.

Category	GO ID	Term	Count	*p* Value
Biological process	GO:0043066	Negative regulation of apoptotic process	4	0.01
	GO:0009812	Flavonoid metabolic process	2	0.01
	GO:0042493	Response to drug	3	0.03
	GO:0006446	Regulation of translational initiation	2	0.03
	GO:0034644	Cellular response to UV	2	0.04
Cell component	GO:0031012	Extracellular matrix	4	0.001
	GO:0005739	Mitochondrion	6	0.002
	GO:0005829	Cytosol	8	0.01
	GO:0070062	Extracellular exosome	7	0.01
	GO:0005925	Focal adhesion	3	0.04
Molecular function	GO:0005515	Protein binding	13	0.003
	GO:0044822	Poly(A) RNA binding	5	0.009
	GO:0016491	Oxidoreductase activity	3	0.01

**TABLE 2:** Gene ontology enrichment analysis for idiopathic cardiomyopathy.

Category	GO ID	Term	Count	*p* Value
Biological process	GO:0044344	Cellular response to fibroblast growth factor stimulus	3	<0.001
	GO:0043066	Negative regulation of apoptotic process	5	0.001
	GO:0042493	Response to drug	4	0.003
	GO:0043488	Regulation of mRNA stability	3	0.004
	GO:0009812	Flavonoid metabolic process	2	0.01
	GO:0000289	Nuclear-transcribed mRNA poly(A) tail shortening	2	0.03
	GO:0071364	Cellular response to epidermal growth factor stimulus	2	0.03
	GO:0006446	Regulation of translational initiation	2	0.03
	GO:0034644	Cellular response to UV	2	0.04
	GO:0006631	Fatty acid metabolic process	2	0.05
Cell component	GO:0005829	Cytosol	12	<0.001
	GO:0005739	Mitochondrion	7	0.001
	GO:0042470	Melanosome	3	0.004
	GO:0005634	Nucleus	11	0.01
	GO:0070062	Extracellular exosome	7	0.03
	GO:0031012	Extracellular matrix	3	0.03
	GO:0005925	Focal adhesion	3	0.05
	GO:0005886	Plasma membrane	8	0.05
Molecular function	GO:0005515	Protein binding	16	<0.001
	GO:0044822	Poly(A) RNA binding	6	0.002
	GO:0016491	Oxidoreductase activity	3	0.01
	GO:0008017	Microtubule binding	3	0.01
	GO:0019901	Protein kinase binding	3	0.04

**TABLE 3:** Kyoto Encyclopedia of Genes and Genomes pathway analysis for ischemic cardiomyopathy and idiopathic cardiomyopathy.

	Term	Count	*p* Value
Ischemic cardiomyopathy	MAPK signaling pathway	3	0.04
	HTLV-I infection	3	0.04
Idiopathic cardiomyopathy	HTLV-I infection	4	0.01
	PI3K-Akt signaling pathway	4	0.02
	Hepatitis B	3	0.02
	Hippo signaling pathway	3	0.02

### Protein-Protein Interaction Network Construction and Hub Genes Analysis

**FIGURE 3:**
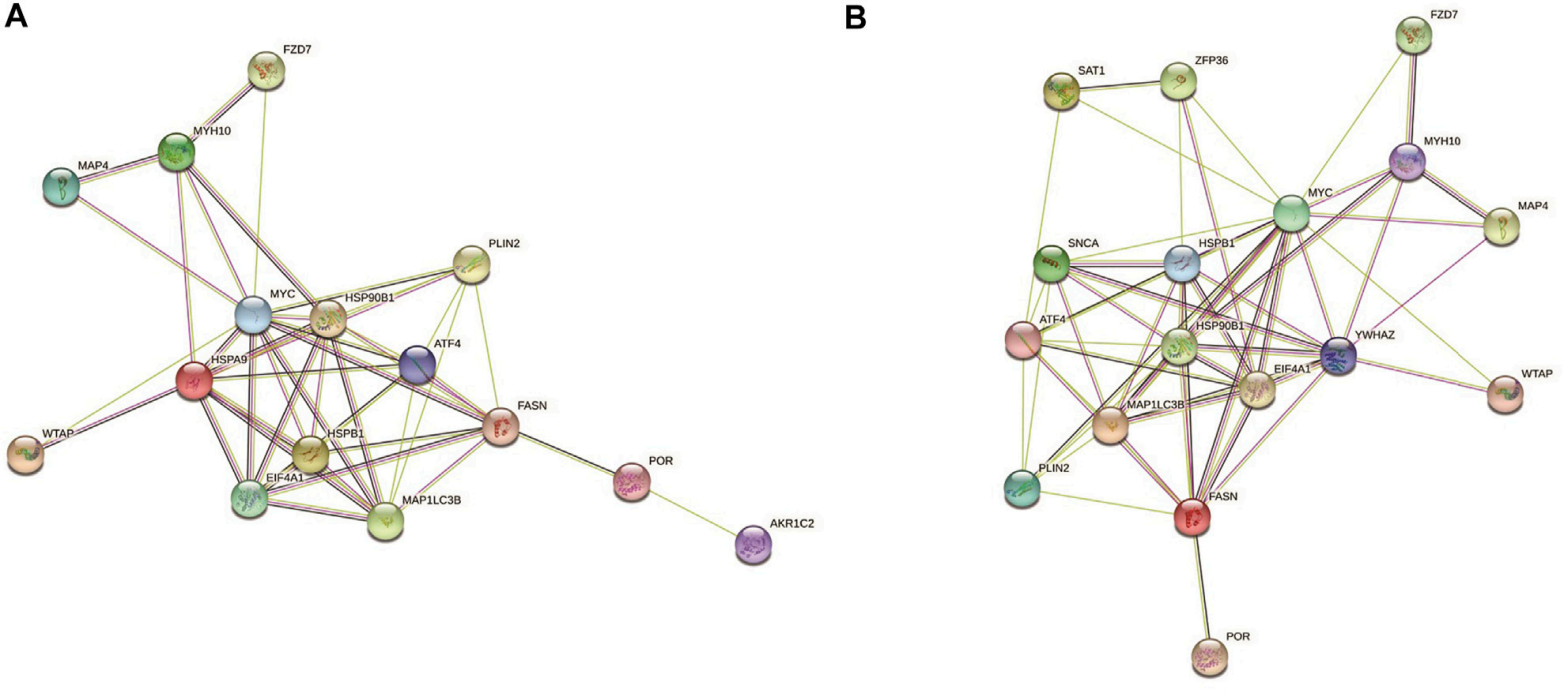
The protein–protein interaction network constructed via STRING database representing the degree of gene interaction. **(A)** ischemic cardiomyopathy. **(B)** idiopathic cardiomyopathy.

**FIGURE 4:**
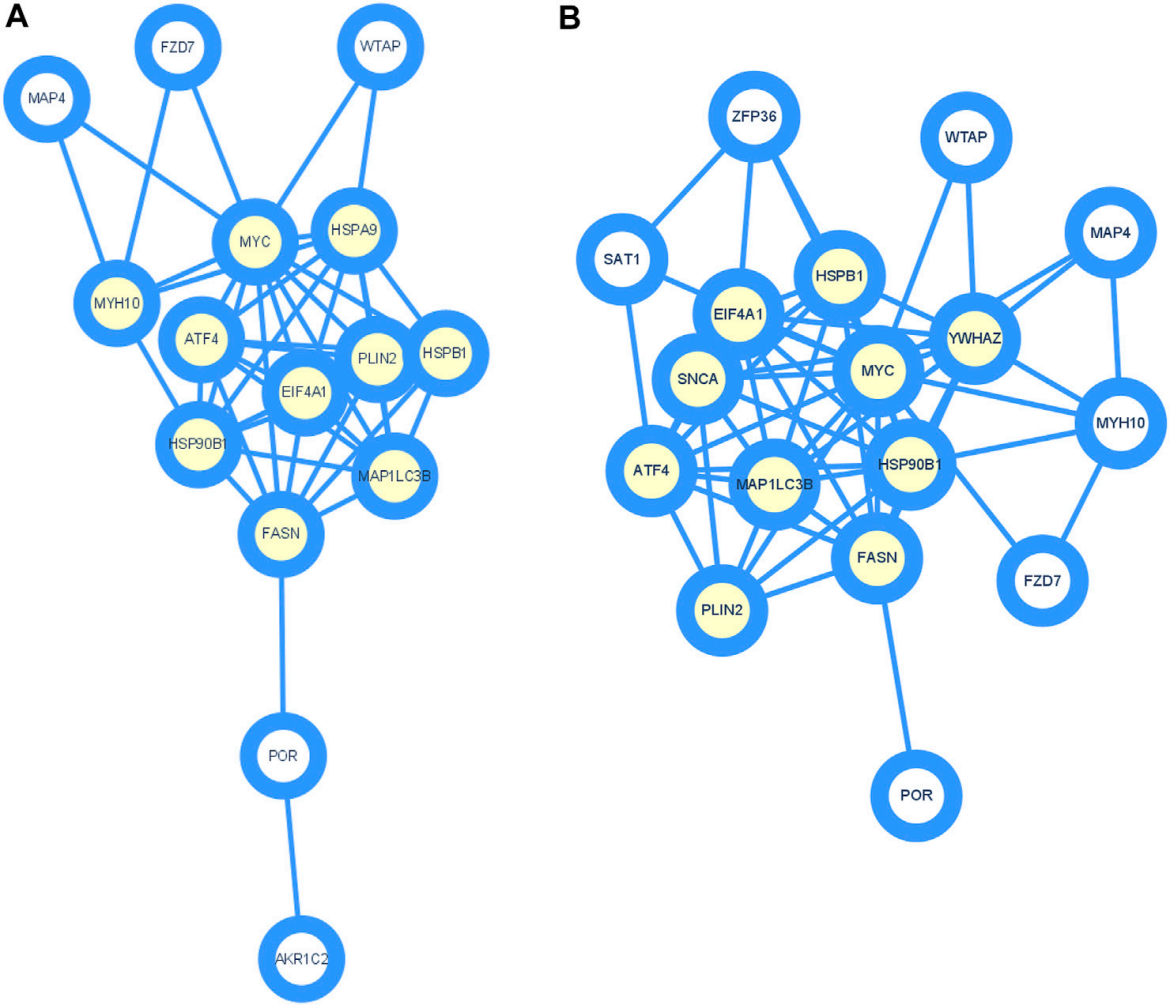
Identification of hub genes using Cytoscape and MCODE plugin. **(A)** ischemic cardiomyopathy. **(B)** idiopathic cardiomyopathy.

To further study the roles of ferroptosis related DEGs, we used protein-protein interaction data from STRING to construct a network containing 15 nodes and 44 edges for ischemic cardiomyopathy (Figure 3A) and 17 nodes and 56 edges for idiopathic cardiomyopathy (Figure 3B). We identified a potential key module and generated a co-expression network for it, consisting of 10 nodes and 36 edges for ischemic cardiomyopathy (Figure 4A) and 10 nodes and 39 edges for idiopathic cardiomyopathy (Figure 4B). The proteins in the network mainly function in cellular response to unfolded protein, response to unfolded protein and cellular response to organic substance for ischemic cardiomyopathy and dopamine uptake involved in synaptic transmission, dopamine biosynthetic process and dopamine metabolic process for idiopathic cardiomyopathy.

### Screening for Potential Pharmacological Targets

Screening results of potential pharmacological targets were downloaded from cMAP, ranked and filtered based on connectivity scores for drugs. Top 10 medications suggested for ischemic cardiomyopathy were QW-BI-011, SA-792709, caffeic-acid, capsazepine, ritonavir, CAY-10618, econazole, alvespimycin, WR-216174, and diphencyprone (Table 4). Top 10 medications suggested for idiopathic cardiomyopathy were varenicline, RO-90-7501, erythrosine, tofacitinib, fasudil, rucaparib, VX-702, nisoxetine, lobaric-acid, and spironolactone (Table 5). These drugs are potential to be a therapeutic agent for ischemic/idiopathic cardiomyopathy.

**TABLE 4:** Top 10 prediction results from cMap for ischemic cardiomyopathy.

Score	Name	Description
−99.86	QW-BI-011	Histone lysine methyltransferase inhibitor
−99.82	SA-792709	Retinoid receptor agonist
−99.79	caffeic-acid	Lipoxygenase inhibitor
−99.77	capsazepine	TRPV agonist
−99.75	Ritonavir	HIV protease inhibitor
−99.75	CAY-10618	NAMPT inhibitor
−99.75	econazole	Bacterial cell wall synthesis inhibitor
−99.72	alvespimycin	HSP inhibitor
−99.68	WR-216174	PFMRK inhibitor
−99.67	diphencyprone	Immunostimulant

**TABLE 5:** Top10 prediction results from cMap for idiopathic cardiomyopathy.

Score	Name	Description
−99.93	varenicline	Acetylcholine receptor agonist
−99.93	RO-90-7501	Beta amyloid inhibitor
−99.93	erythrosine	Coloring agent
−99.93	Tofacitinib	JAK inhibitor
−99.89	fasudil	Rho associated kinase inhibitor
−99.89	rucaparib	PARP inhibitor
−99.86	VX-702	p38 MAPK inhibitor
−99.86	nisoxetine	Norepinephrine reuptake inhibitor
−99.86	lobaric-acid	Tyrosine phosphatase inhibitor
−99.82	spironolactone	Mineralocorticoid receptor antagonist

## Discussion

Cardiomyopathies are a heterogeneous group of diseases that can be divided into two categories, primary and secondary, and are common disorders causing heart failure and sudden cardiac death. Ischaemic cardiomyopathy is the most common type of secondary cardiomyopathy and is mostly caused by coronary multivessel disease or even diffuse lesions causing extensive ischaemia, degeneration, necrosis and fibrosis of the myocardium, additionally doped with myocardial stunning and hibernation, which leads to severe myocardial dysfunction, spherical enlargement of the heart, and (or) heart failure (Li et al., 2019). Idiopathic cardiomyopathies are cardiomyopathies caused by genetic, non-genetic, and acquired etiologies, singly or mixed. The pathogenesis of ferroptosis in ischemic and idiopathic cardiomyopathy is poorly understood, and the key genes and pathways associated with ferroptosis in cardiomyopathy await further discovery (Lee et al., 2020). In this study, we first obtained ferroptosis related differentially expressed genes of ischemic/idiopathic cardiomyopathy by analyzing the gene expression profiles of ischemic/idiopathic cardiomyopathy and healthy patients. A total of 15 (4 upregulated, 11 downregulated) differentially expressed genes of ischemic cardiomyopathy, 17 (13 upregulated, 4 downregulated) of idiopathic cardiomyopathy associated with ferroptosis were unearthed, which illustrated that ferroptosis related genes were involved in the pathogenesis of ischemic/idiopathic cardiomyopathy, prompting potential pharmacological targets. To understand the roles of these ferroptosis related genes in ischemic/idiopathic cardiomyopathy, we further performed GO enrichment analysis and found that the biological processes mainly involved in negative regulation of apoptotic process, flavonoid metabolic process, response to drug, regulation of translational initiation, and cellular response to UV for ischemic cardiomyopathy, which was consistent with previous studies. There is ample evidence that flavonoids have beneficial effects on myocardial ischemia-reperfusion *in vitro*, which is promising as a treatment of chronic conditions such as ischemic heart disease (Yuan et al., 2015). For idiopathic cardiomyopathy, the biological processes involved in these genes mainly include cellular response to fibroblast growth factor stimulus, negative regulation of apoptotic process. Myocardial fibrosis is now widely recognized as an important part of the pathogenesis of cardiac remodeling and progression to heart failure. Cardiac fibroblast activation promotes the accumulation of collagen types I and III, which are major fibrin of the myocardial collagen matrix. The structural remodeling of cardiac interstitium is a major determinant of pathological hypertrophy (Kumbar et al., 1985). Targeted inhibition of ferroptosis may be a new direction for the treatment of ischemic/idiopathic cardiomyopathy.

KEGG pathway analysis found that the differentially expressed genes related to ferroptosis in ischemic cardiomyopathy were mainly involved in MAPK signaling pathway. MAPK, a stress activated protein kinase, regulates ischemia-reperfusion cardiomyocyte apoptosis through single protein kinase activity (Zhu et al., 2021). The differentially expressed genes related to ferroptosis in idiopathic cardiomyopathy were mainly involved in PI3K-Akt signaling pathway. PI3K-Akt is an important signal transduction pathway in the regulation of cardiac function, mainly through vascular endothelial growth factor mediated angiogenesis, inhibition of cardiomyocyte apoptosis, remodeling of the ventricle, and promotion of cellular energy metabolism (Wang et al., 2021). These provide evidence for targeted therapy of ischemic/idiopathic cardiomyopathy.

We also constructed a protein-protein interaction network of ferroptosis related differentially expressed genes by STRING (Figure 3), and 10 hub genes for ferroptosis in ischemic cardiomyopathy: HSPA9, FASN, HSPB1, EIF4A1, ATF4, MAP1LC3B, HSP90B1, MYC, PLIN2, MYH10, and were identified by Cytoscape (Figure 4A). HSPA9 (Heat Shock Protein Family A (Hsp70) Member 9) is an important apolipoprotein interacting protein. HSPA9 overexpression prevents the import of apoptotic proteins into the nucleus for proapoptotic activity (Gholizadeh et al., 2020). FASN (Fatty Acid Synthase)-mediated lipid metabolism contributes to regulating apoptosis and steroidogenesis (Liu et al., 2018). HSPB1 (Heat Shock Protein Family B (Small) Member 1) is a small heat shock protein which probably maintains denatured proteins in a folded state as a molecular chaperone (Wang et al., 2019). EIF4A1 (Eukaryotic Translation Initiation Factor 4A1) is a Protein Coding gene which related to nucleic acid binding and hydrolase activity (Steinberger et al., 2020). ATF4 (Activating Transcription Factor 4) is involved in DNA-binding transcription factor activity and protein heterodimerization activity (Kasetti et al., 2020). MAP1LC3B (Microtubule Associated Protein 1 Light Chain 3 Beta) regulates mitophagy by eliminating mitochondria to basal levels to meet cellular energy demands and prevent excessive ROS production, contributing to the optimization of mitochondrial quantity and quality (Hayashi et al., 2020). HSP90B1 (Heat Shock Protein 90 Beta Family Member 1) is associated with calcium ion binding and unfolded protein binding (Husain et al., 2021). MYC (MYC Proto-Oncogene, BHLH Transcription Factor) is a proto-oncogene and encodes a nuclear phosphoprotein that functions in cell cycle progression, apoptosis, and cell transformation (Flynn and Hogarty, 2018). PLIN2 (Perilipin 2) is associated with the lipid globule surface membrane material that may be involved in adipose tissue development and maintenance (Bildirici et al., 2018). Mutations in MYH10 (Myosin Heavy Chain 10) have been associated with May-Hegglin anomaly and developmental defects in brain and heart (Schliffka et al., 2021). Ten hub genes: YWHAZ, EIF4A1, FASN, HSPB1, ATF4, HSP90B1, MYC, MAP1LC3B, PLIN2, SNCA, and were identified for ferroptosis in idiopathic cardiomyopathy by Cytoscape (Figure 4B). Among them, EIF4A1, FASN, HSPB1, ATF4, HSP90B1, MYC, MAP1LC3B, and PLIN2 were the same as ischemic cardiomyopathy. YWHAZ (Tyrosine 3-Monooxygenase/Tryptophan 5-Monooxygenase Activation Protein Zeta) interacts with IRS1 protein, contributing to regulation of insulin sensitivity (Popov et al., 2019). SNCA (Synuclein Alpha) may serve to integrate presynaptic signaling and membrane trafficking (Tagliafierro et al., 2019). These ferroptosis related hub genes may be potential therapeutic targets and require further investigation.

Notably, in this study, we first screened the differential expressed genes related to cardiomyopathy, and picked out those related to ferroptosis from the differential expressed genes. The genes related to ferroptosis in healthy people (that is to say, genes related to ferroptosis but not related to cardiomyopathy) were not included in the analysis. The benefit of this is that the analysis of ferroptosis-related differential expressed genes are disease-specific. Targeted therapy against these genes may be more helpful to improve the prognosis of cardiomyopathy.

There are several limitations in this study. These ferroptosis related genes differentially expressed in ischemic and idiopathic cardiomyopathy, especially the hub genes and signaling pathways, which need to be further verified by cell experiments, and clinical samples. In addition, we screened out potential therapeutic compounds targeting hub genes and pathways by bioinformatics methods, which provided a certain theoretical basis for exploring new therapeutic strategies, but further verification by animal experiments is required.

## Conclusion

In conclusion, the results of this study reveal the potential role of ferroptosis in ischemic and idiopathic cardiomyopathy, and find the hub genes and pathways involved by bioinformatics methods, which may provide a theoretical basis for exploring new therapeutic strategies. Further studies are needed in the future to explore the causal relationship of ferroptosis with ischemic and idiopathic cardiomyopathies, which may provide novel potential therapeutic targets and prognostic markers.

## Data Availability

Publicly available datasets were analyzed in this study. This data can be found here: https://www.ncbi.nlm.nih.gov/geo/query/acc.cgi?acc=GSE5406.
