# Supplementary material for: Ferroptosis Related Genes in Ischemic and Idiopathic Cardiomyopathy: Screening for Potential Pharmacological Targets
Source: Front Cell Dev Biol. 2022 Mar 3;10:817819. doi: 10.3389/fcell.2022.817819 (PMC8927736; doi:10.3389/fcell.2022.817819)
Supplement: Supplementary file 1 [file Table2.DOCX]

Supplementary Table 2. Analysis of the dysregulated genes in ischemic cardiomyopathy

| Dysregulation | Gene | Description |
| --- | --- | --- |
| Upregulated | MAP4 | promotes microtubule assembly, and has been shown to counteract destabilization of interphase microtubule catastrophe promotion |
|  | FZD7 | downregulates APC function and enhance beta-catenin-mediated signals |
|  | MYH10 | associated with May-Hegglin anomaly and developmental defects in brain and heart |
|  | POR | encodes an endoplasmic reticulum membrane oxidoreductase that is essential for multiple metabolic processes, including reactions catalyzed by cytochrome P450 proteins for metabolism of steroid hormones, drugs and xenobiotics |
| Downregulated | HSPB1 | plays an important role in the differentiation of a wide variety of cell types |
|  | FASN | catalyzes the synthesis of palmitate from acetyl-CoA and malonyl-CoA, in the presence of NADPH, into long-chain saturated fatty acids. |
|  | HSPA9 | plays a role in cell proliferation, stress response and maintenance of the mitochondria |
|  | EIF4A1 | related pathways are Interferon gamma signaling and Transport of the SLBP independent Mature mRNA |
|  | PLIN2 | associated with the lipid globule surface membrane material, and maybe involved in development and maintenance of adipose tissue. |
|  | HSP90B1 | associated with a variety of pathogenic states, including tumor formation. |
|  | AKR1C2 | encodes a member of the aldo/keto reductase superfamily, which consists of more than 40 known enzymes and proteins |
|  | WTAP | plays a role in both transcriptional and posttranscriptional regulation of certain cellular genes. Alternative splicing of this gene results in several transcript variants encoding three different isoforms. |
|  | MAP1LC3B | related pathways are Autophagy pathway and Cellular Senescence |
|  | MYC | plays a role in cell cycle progression, apoptosis and cellular transformation |
|  | ATF4 | encodes a transcription factor that was originally identified as a widely expressed mammalian DNA binding protein that could bind a tax-responsive enhancer element in the LTR of HTLV-1 |
